# Higher Inflammatory Response in Hepatocellular Carcinoma is Associated with Immune Cell Infiltration and a Better Outcome

**DOI:** 10.21203/rs.3.rs-3768964/v1

**Published:** 2024-01-03

**Authors:** Masanori Oshi, Kohei Chida, Arya Mariam Roy, Gabriella Kim Mann, Nan An, Li Yan, Itaru Endo, Kazuaki Takabe

**Affiliations:** Roswell Park Comprehensive Cancer Center; Roswell Park Comprehensive Cancer Center; Roswell Park Comprehensive Cancer Center; Roswell Park Comprehensive Cancer Center; Roswell Park Comprehensive Cancer Center; Roswell Park Comprehensive Cancer Center; Yokohama City University: Yokohama Shiritsu Daigaku; Roswell Park Comprehensive Cancer Center

**Keywords:** Biomarker, Carcinogenesis, Hepatocellular Carcinoma, Inflammatory

## Abstract

**Background & Aims:**

Hepatocellular carcinoma (HCC) often develops from chronic liver inflammation. Inflammation within a tumor can either promote cancer progression or activate an immune response against it. This study aims to determine the clinical significance of enhanced inflammation in HCC.

**Methods:**

Data from 655 HCC patients across four cohorts (TCGA, GSE6764, GSE76427, GSE89377) were examined. Inflammatory response was quantified using a scoring system derived from the gene set variation analysis of the “INFLAMMATORY_RESPONSE” gene set.

**Results:**

A stepwise increase in inflammatory response was noted from normal liver to cirrhosis, with consistently lower levels in HCC across both GSE6764 and GSE89377 cohorts (both *p*<0.001). Similar trends were observed in interferon response, pathways such as IL6/JAK/STAT3 and complement signaling, coagulation cascade, and allograft rejection (all *p*<0.02). HCCs with high inflammatory response were associated with increased immune cell infiltrations (*p*<0.01) and cytolytic activity (*p*<0.001). Interestingly, these HCCs had reduced mutation rates, no relationship with cell proliferation, and displayed both immune responses and pro-cancerous signals including epithelial-mesenchymal transition, KRAS, and hypoxia. Further, a high inflammatory score correlated with improved disease-free survival in TCGA (*p*=0.034) and overall survival in GSE76427 (*p*=0.008).

**Conclusion:**

HCC with higher levels of inflammatory response demonstrated increased immune cell infiltration, enhanced immune-related and other pro-cancerous-related signaling, and better patient prognosis.

## 1. Introduction

Liver cancer is the eighth most common cancer and the third leading cause of cancer-related mortality globally, and 80% of them are hepatocellular carcinoma (HCC) [1]. The majority of HCC cases stem from chronic liver inflammation, primarily associated with conditions such as fatty liver disease, hepatitis virus infections, and exposure to toxic substances, including alcohol. Chronic liver damage by necro-inflammation has been shown to lead to persistent hepatocyte regeneration, resulting in genetic mutations in hepatocytes and the proliferation of abnormal cells that become HCC [2]. These can progress to liver cirrhosis and, in some cases, develop into HCC.

Chronic inflammation is widely recognized for its significant role in carcinogenesis across various cancers. Chemicals and cytokines released by inflamed cells, including reactive oxygen species, can damage nearby cells, accelerating their genetic evolution toward more malignant states. Rudolf Virchow observed the relationship between inflammation and carcinogenesis as early as the nineteenth century [3] and described leukocyte infiltrates within tumors, which are now considered one of the Hallmarks of Cancer [4]. Currently chronic inflammation is estimated to contribute to 15 to 25% of human cancers [5]. For instance, infections by helicobacter pylori, papillomaviruses, hepatitis viruses, inflammatory conditions of uncertain origin or some autoimmune diseases, are known to cause chronic inflammation, thereby increasing the risk of specific cancers like that increases the risk of cancer such as hepatitis for HCC, prostatitis for prostate cancer, and inflammatory bowel disease for colon cancer [6]. Further, chronic inflammation has been shown to promote cancer progression, in several type of cancers. Inflammatory mediators can directly interact with cancer cells and stromal cells and promote epithelial-to-mesenchymal transition (EMT) that leads to cancer cell metastasis [7]. Chronic inflammation has been shown to contribute to multiple Hallmarks of Cancer, including genomic instability, proliferative signaling, and immune system evasion [4].

It is essential to comprehend the factors influencing carcinogenesis and cancer progression, as some of these factors may exert divergent effects during development and advancement of cancer [8]. Carcinogenesis is the initial phase of cancer, characterized by mutations that lead to abnormal cellular proliferation and the formation of a tumor cluster. In contrast, cancer progression signifies the exacerbation of an existing cancer, marked by gene mutations that contribute to intratumoral genomic heterogeneity and occurrence of metastasis [8]. Notably, certain cellular pathways exert opposing effects during these two processes. DNA repair mechanisms, for instance, are crucial inhibiting carcinogenesis, while the same processes can give established cancer cells an advantage by enabling them to resist cytotoxic therapies and perpetuate genetic instability. While chronic inflammation is well-known to be carcinogenic, its effects on the progression of already established HCCs remain unclear.

Previously, we utilized gene set variation analysis (GSVA) on the transcriptome of bulk tumors to explore the impact of signaling networks in various malignancy types including HCC [9–11]. GSVA scores, estimating pathway activation levels by analyzing numerous genes associated with a given pathway[12], were calculated. We specified the inflammatory response score as the GSVA score derived from the “INFLAMMATORY_RESPONSE” gene set in the molecular signatures database (MSigDB. Our results revealed that inflammation was associated with adverse outcomes within the entire breast cancer cohort. Interestingly, this association was inverted for patients with triple-negative breast cancer, where inflammation was linked to improved outcomes [13, 14]. Therefore, we extended our investigation to evaluate the clinical relevance of high inflammation in patients with HCC using this score.

## 2. Materials and Methods

### 2.1. Data acquisition o HCC

A total of 655 HCC samples with mRNA expression and clinicopathological data were obtained from multiple publicly accessible data bases including the Cancer Genome Atlas (TCGA; *n* = 358) [15] and GEO database (GSE6764 (*n* = 75) [16], GSE76427 (*n* = 115) [17], and GSE89377 (*n* = 107) [18] cohorts).

### 2.2. Inflammatory response signaling score

GSVA scoring [12] with the “HALLMARK_INFLAMMATORY_RESPONSE” gene set that includes 200 inflammatory-related genes in the MSigDB gene sets collection [14] was used to quantify the inflammatory response, which is the same score we used to analyze breast cancer cohort [13]. We employed the median as a cut-off to categorize the cohorts into groups with low and high inflammatory scores.

### 2.3. Biological function analysis

In order to investigate the biological functions related to Hallmarks of cancer associated with a high inflammatory response in HCC within each cohort, we employed a gene set enrichment analysis (GSEA) algorithm [19]. The statistical significance was defined as a false discovery rate (FDR) of less than 25%, as recommended by GSEA [20]. As reported earlier in our prior articles [21, 22], we obtained the Hallmark cancer gene sets from the hallmark gene set collection within MSigDB [14]

### 2.3. Other scores

We used the CYT score to assess the cytolytic activity, which is calculated as the geometric mean of granzyme A (GZMA) and perforin (PRF1) expression in Transcripts Per Million (TPM) [23]. An xCell algorithm was then employed to estimate the fraction of several immune and stromal cell types in tumor tissues, evaluating intra-tumor microenvironment composition [24]. Thorsson et al. provided additional score values for TCGA samples, including intratumor heterogeneity, homologous recombination defects, fraction altered, insertions and deletions (indel) neoantigens, single-nucleotide variant (SNV) neoantigens, silent and non-silent mutations [25].

### 2.4. Statistical analysis

This study was analyzed using R software (version 4.1.0). Boxplots depict medians and interquartile range (IQR). For group comparison analysis, we employed the Kruskal-Wallis and Mann-Whitney U tests. Survival analysis was performed used log-rank test and Cox proportional hazards.

## 3. Results

### 3.1. Inflammatory response showed an increase from dysplastic nodule to cirrhosis, but was lower in HCC within the carcinogenic sequence

**Figure 1:**
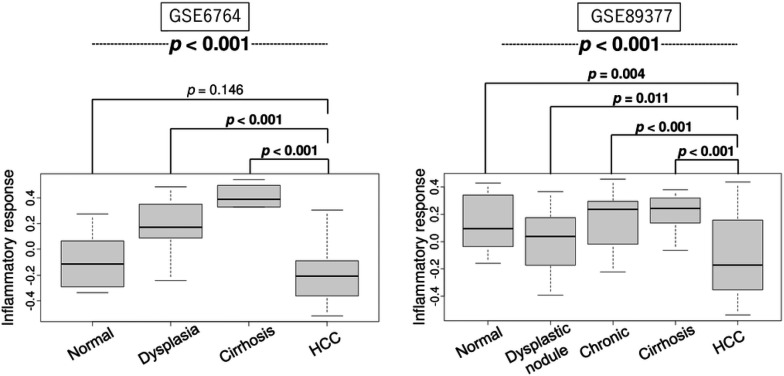
The inflammatory response levels in liver tissues during carcinogenic sequence of HCC. Boxplots of inflammatory response score by normal liver (Normal, n = 10), dysplastic nodule (Dysplasia, *n* = 17), Cirrhosis (*n* = 13), and HCC (*n* = 35) in the GSE6764 cohort, and by Normal (*n* = 13), Dysplastic nodule (*n*= 22), Chronic hepatitis (*n* = 20), Cirrhosis (*n* = 12), and HCC (*n*= 40) in the GSE89377 cohort. P-value was calculated by Kruskal-Wallis and Mann-Whitney U test.

Since chronic inflammation plays a critical role in the carcinogenesis of HCC, we first investigated the levels of inflammatory response in liver tissues; normal, dysplastic nodule, chronic hepatitis, cirrhosis and HCC, also known as HCC carcinogenic sequence. We found that the inflammatory response level increased during the stepwise carcinogenic process until cirrhosis (Fig. 1). Interestingly, inflammatory response was decreased in HCC compared to other states constantly in both the GSE6764 and GSE89377 cohorts (Fig. 1, both *p* < 0.001). This result implies that while the inflammatory response was heightened leading up to the onset of cancer, its activity wanes once the cancer was established.

### 3.2. Multiple immune response-related signaling follow the same trend as the inflammatory response in the HCC carcinogenesis sequence

**Figure 2:**
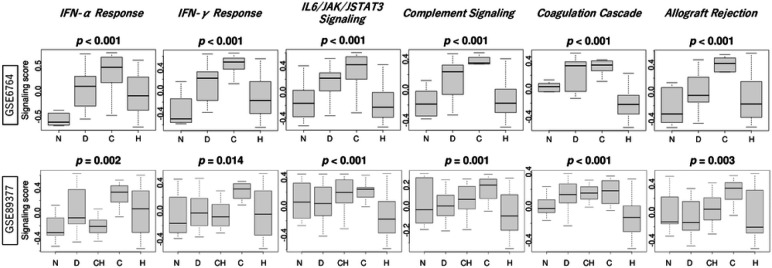
The activation of immune response-related pathways in liver tissues during the carcinogenic sequence of HCC. Boxplots of immune response-related gene set scores, interferon(IFN)-α, γ response, complement signaling, IL6/JAK/STAT3 signaling, coagulation cascade, and allograft rejection, by normal liver (N, *n* = 10), Dysplasia (D, *n* = 17), Cirrhosis (C, *n* = 13), and HCC (H, *n* = 35) in the GSE6764 cohort, and by Normal liver (N, *n* = 13), Dysplastic nodule (D, *n* = 22), Chronic Hepatitis (CH, *n* = 20), Cirrhosis (C, *n* = 12), and HCC (H, *n* = 40) in the GSE89377 cohort. Kruskal-Wallis test was used to determine p-value.

Given that inflammatory response elevated with the stepwise carcinogenic process, we then investigated the association of carcinogenic process with several immune response-related pathways defined by the MSigDB [14], including interferon (IFN)-α and IFN-γ responses, complement signaling, IL6/JAK/STAT3 signaling, coagulation cascade, and allograft rejection. We found that all these immune response-related pathways exhibited similar trends to the inflammatory response, increasing during the stepwise carcinogenic process until cirrhosis but decreasing in HCC, often reaching levels lower than or close to those in normal liver consistently in GSE6764 and GSE89377 cohorts (Fig. 2, all *p* < 0.02). These results suggest that multiple immune response-related signaling pathways follow a comparable trend to the inflammatory response within the carcinogenesis sequence of HCC.

### 3.3. Correlation of immune cell infiltration and inflammatory response levels in HCC tumor millieu

**Figure 3:**
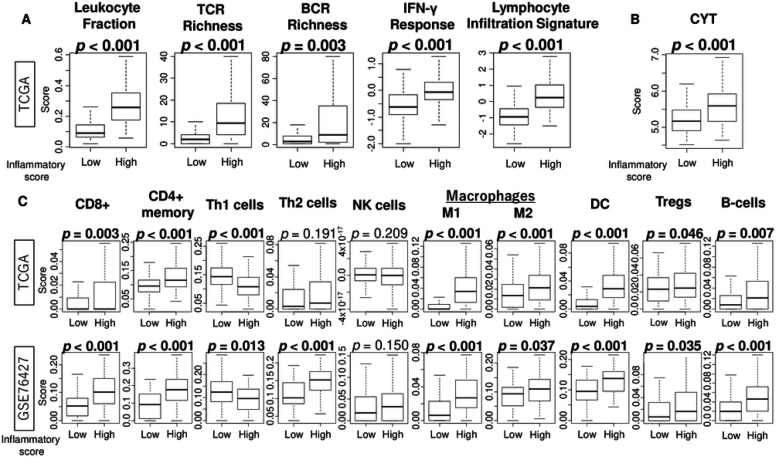
The immunity-related scores and infiltration fractions of immune cells in tumor microenvironment by the inflammatory response in HCC. Boxplots of (A) immunity-related score; T cell receptor (TCR) richness and B cell receptor (BCR) richness, leukocyte fraction, interferon (IFN)-γ response, and lymphocyte infiltration signature, and (B) cytolytic activity (CYT), and (C) infiltration fractions of CD8^+^ T cells, CD4^+^ memory T cells, helper T type 1 (Th1) and type 2 (Th2) cells, NK cells, M1 and M2 macrophages, and dendritic cell (DC), regulatory T cells (Tregs), and B-cells by low and high inflammatory response groups in TCGA and GSE76427 cohorts. Mann-Whitney U test was used to calculate the significant difference between two groups.

Given the notably lower inflammatory response observed in HCC compared to other liver tissues within the carcinogenesis sequence, we were intrigued to explore the relationship of inflammatory response and immune cell infiltration in HCC. First we analyzed the immunity-related scores precalculated on TCGA samples by Thorsson et al. [25], which included richness of T-cell and B-cell receptors (TCR and BCR), leukocyte fraction, IFN-γ response, and lymphocyte infiltration signature scores. High inflammatory response HCC was found to be linked with higher levels of all immunity-related scores (Fig. 3A, all *p* ≦ 0.003), as well as a significantly high level of cytolytic activity (CYT) in TCGA (Fig. 3B, p < 0.001). Next, we compared the infiltrating fractions of immune cells by the inflammatory response and found that in the TCGA and GSE6427 cohorts, high inflammatory response HCC was associated with significantly high infiltrations of many immune cells, including CD8^+^, CD4^+^ memory, M1 and M2macrophages, dendritic cells, regulatory T cells (Tregs), and as well as B cells, and with less infiltrations of helper T type 1 cells (Fig. 3C). There were no consistent validated findings in the other cells. These results suggest that although the overall levels of inflammatory response are low in HCC compared to the other conditions within the carcinogenesis sequence, higher inflammatory response among HCC is linked with higher infiltrations of multiple immune cellular components compared to the lower inflammatory response HCC groups.

### 3.4. HCC exhibiting a high inflammatory response showed a significant correlation with low mutation rates and neoantigen levels, while no relationship was found with cell proliferation

**Figure 4:**
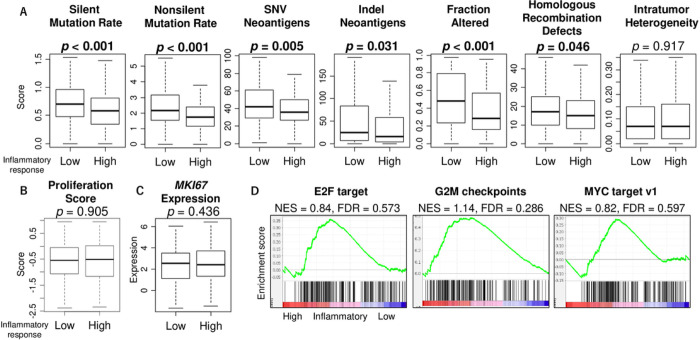
Association of the inflammatory response with mutation rates, neoantigens, fraction altered, homologous recombination deficiency, and intratumor heterogeneity, as well as cell proliferation-related signatures. Boxplots of (A) homologous recombination defects (HRD), intratumor heterogeneity, and the mutation-related scores; single nucleotide variation (SNV), fraction altered, silent and non-silent mutation rate, and indel neoantigens and cell proliferation-related factors; (B) proliferation score and (C) Ki67 gene (*MKI67*) expression, by low and high inflammatory response score groups. Mann-Whitney U test was used to calculate p-value. (D) Enrichment plots for gene sets associated with cell proliferation, such as E2F target, MYC target v1, and G2M checkpoints based on the inflammatory response scores categorized into low and high groups. The score groups were segmented using the median value as a cut-off.

Through neoantigens resulting from high tumor mutational burden, cancer cells are known to attract tumor-infiltrating immune cells [26]. Simultaneously, highly mutated cancers are known to be proliferative and aggressive [26, 27]. Therefore, we investigated the association of inflammatory response with mutation rate and cell proliferation. Interestingly, we observed that high inflammatory response was linked with significantly low levels of silent and non-silent mutation rate, fraction altered, homologous recombination defects (HRD), intratumor heterogeneity, and single nucleotide variation (SNV) and indel neoantigens (Fig. 4A). This result was somewhat opposite from what we expected from immune cell infiltrations. Next, we investigated the relationship between inflammatory response and cell proliferation in HCC, using proliferation score and Ki67 gene (*MKI67*) expression. Despite the significantly lower mutation rate in HCC with high inflammatory response, there was no significant difference in the marker of cell proliferation (Fig. 4B and 4C, p = 0.905 and 0.436, respectively). In agreement, E2F target, G2M checkpoints, and MYC target v1, which are the cell proliferation gene sets, did not demonstrate any enrichment to inflammatory response (Fig. 4D). These results suggest that inflammatory response was inversely related to mutation rates and neoantigens but showed no relationship with cell proliferation in HCC.

### 3.5. High inflammatory response HCC enriched not only immune response-related but also several pro-cancerous signaling

**Figure 5:**
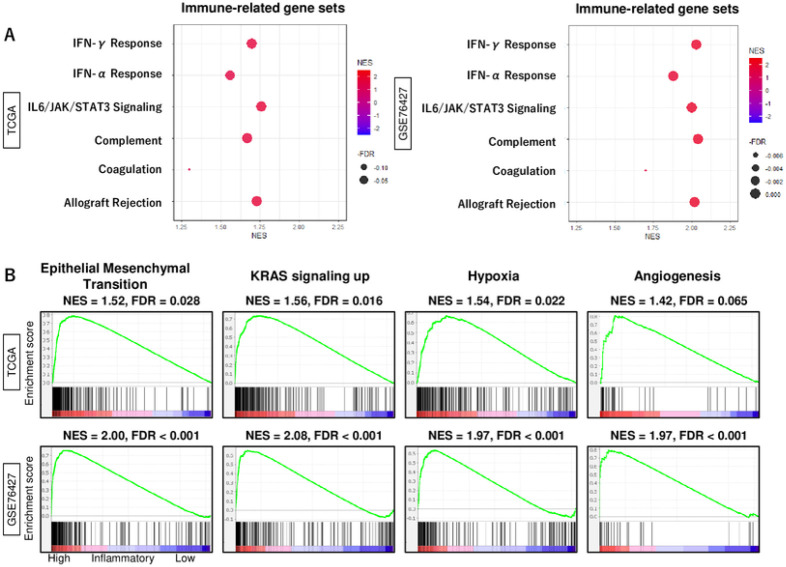
The biological function features of high inflammatory response score in HCC. (A) Dot plots of interferon (IFN)-α and -γ response, Complement, Coagulation, and allograft rejection, IL6/JAK/STAT3 signaling in both patient cohorts from TCGA and GSE76427 by GSEA. (B) Enrichment plots of pro-cancerous signaling; KRAS signaling up, apoptosis, epithelial mesenchymal transition, hypoxia, angiogenesis, which were enriched significantly in the HCC with enhanced inflammatory response score. This enrichment was observed consistently in both cohorts through GSEA. As recommended by the GSEA software, FDR < 0.25 defined statistical significance. NES, normalized enrichment score; FDR, false discovery rate.

We then investigated which other pathways could be associated with the inflammatory response in HCC. In our analysis, we observed a significant enrichment of all immune-related gene sets, including interferon (IFN)-α, IFN-γ response, IL6/JAK/STAT3 signaling, Complement, Coagulation, and allograft rejection, in HCC with a high inflammatory response. This enrichment was consistent in both TCGA and GSE76427 cohorts (Fig. 5A). Additionally, we found an enrichment of multiple pro-cancerous gene sets, such as KRAS signaling up, Epithelial Mesenchymal Transition (EMT), hypoxia, and angiogenesis, consistently in these cohorts. This denotes that HCC with a high inflammatory response exhibit an enhanced immune response as well as activation of cancer-promoting pathways.

### 3.6. HCC with a high inflammatory response showed a favorable correlation with survival compared to the group with a low score

**Figure 6:**
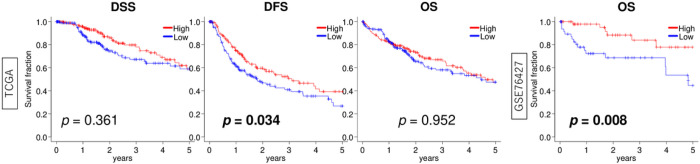
Survival relevance of inflammatory response in HCC. Kaplan-Meier curve with log-rank p-value of disease-specific survival (DSS), disease free survival (DFS), and overall survival (OS) in TCGA and OS in GSE76427 between HCC with low (blue line) and high (red line) inflammatory response scores. We utilized the median value as a threshold to categorize the two score groups into high and low.

Finally, we assessed the clinical importance of inflammatory response in HCC patients. In our findings, the high inflammatory response group exhibited a significant association with improved disease-free survival (DFS) in TCGA (p = 0.034) and overall survival (OS) in the GSE76427 cohort (p = 0.008) (Fig. 6). Although no significant differences were observed between the two groups, the high inflammatory response group tended to exhibit a more favourable patient prognosis than the low-inflammatory group for disease-specific survival (DSS) in the TCGA cohort (Fig. 6, p = 0.361). These results suggest that HCC patients with high inflammatory response score trend to have a better prognosis compared to patients with low score.

## 4. Discussion

In our study, we explored the clinical significance of a heightened inflammatory response in HCC, leveraging the inflammatory response score we had previously computed [13]. We observed that HCC exhibited a decreased level of inflammatory response in comparison with the other liver diseases, even though it increased throughout the carcinogenic process from normal liver to cirrhosis. This trend was mirrored in immune response-related signaling. Within HCC, high inflammatory response tumors had greater immune cell infiltrations. HCCs with high inflammatory response, demonstrated lower mutation rates, neoantigens, HRD and fraction altered. However, there was no relationship observed with cells proliferation gene sets. Moreover, high inflammatory response HCC enriched not only immune response-related pathways but also several pro-cancerous pathways, such as epithelial mesenchymal transition (EMT), KRAS signaling, hypoxia, and angiogenesis. Ultimately, HCC characterized by a robust inflammatory response exhibited superior survival outcomes when contrasted with cases demonstrating a lower score.

Infection and inflammation are well known to promote carcinogenesis and increase cancer risk in certain settings. In HCC, repeated tissue injury caused by chronic exposure to a pathogen can be a primary mechanism for the development of chronic diseases that will eventually promote the development of malignant tumors. There is evidence that inflammation can promote the development of adenoma/neoplasia into invasive cancer [28]. Consequently, inflammation can be viewed as an mediator that adds characteristics of the Hallmarks of Cancer to a normal cell, facilitating its development into cancer [4]. Our study demonstrated that the level of inflammatory response quantified by transcriptomics, increases over time during the carcinogenesis process from normal liver to cirrhosis. Interesting observation is that there was a marked decrease in inflammatory response once HCC is established. Given our approach, we lack data to determine whether our finding suggests that the transformation of cirrhotic tissue to cancer alter the microenvironment in a way that reduces the likelihood of inflammation, or if this is simply because HCC bulk tumor is densely populated with cancer cells allowing relatively less immune cell infiltration that result in less inflammation. On the other hand, among the HCC cases, various immune response-related gene sets were enhanced in high inflammatory response HCCs and both anti- and pro-cancerous immune cells were highly infiltrated compared to with low inflammatory response HCCs. These findings suggest that immune responses may play an important role even in HCC, a status in which inflammatory response level was lower compared to the other liver conditions.

Inflammation and cancer are intricately linked with complicated pathological processes controlled by numerous factors. Nuclear factor kappa B (NF-kB), a transcription factor, has been identified as a key modulator in driving inflammation toward cancer. In addition, an inflammatory microenvironment containing inflammatory cells and a signaling network is essential for the malignant progression of transformed cells [29]. Tumor cells may grow, invade, and metastasize more readily when inflamed tissues are stimulated by inflammatory cells and regulatory molecules. To date, research on inflammation-associated cancer development has predominantly focused on cytokines and chemokines, along with their downstream targets. Moreover, iťs not just NF-kB that plays a role; various signaling pathways, including Janus kinase/signal transducers and activators of transcription (JAK-STAT), toll-like receptor (TLR) pathways, cGAS/STING, and mitogen-activated protein kinase (MAPK), are crucial [30]. Similarly, inflammatory factors such as cytokines, chemokines, growth factors, and inflammasome, along with inflammatory metabolites like prostaglandins, leukotrienes, thromboxane, and specialized resolving mediators (SPM), are essential in regulating the onset and resolution of inflammation [30]. Thus, while numerous hallmark signaling pathways have been identified in the relationship between cancer and inflammation, obtaining a comprehensive understanding of the extent to which each signaling pathway is activated within tumor tissues has proven challenging. Hence, the current study utilized GSVA to examine the entire inflammatory pathway rather than focusing on individual factors. This approach has shown that other immune-related signaling was also enhanced in HCC with high inflammatory response, and that enhanced levels of other pro-cancerous pathway, such as EMT, KRAS, and angiogenesis, were also correlated in the patient’s tumor. HCC inflammation dynamics and their relationship to disease progression may be better understood using this approach.

Cancer progression is the process by which genes mutate and tumor heterogeneity develops [8]. In this study, the tumor mutation burden and neoantigens were examined in depth in order to determine the association of inflammation and immune response in HCC. Our study findings, however, revealed a surprising twist to this general observation. High levels of inflammatory response in HCC were associated with low mutation rates, neoantigens, and homologous recombination defects. This pattern of immune response deviates from the conventional mechanism, suggesting that inflammation and immune responses play an integral part in the process of cancer progression.

Various research studies have demonstrated that chronic inflammation plays an important role in promoting, initiating, and progressing cancer [29]. However, we demonstrate that once HCC develops, the inflammatory response appears to decrease. Conversely, high inflammation is associated with a better prognosis among HCC patients, adding complexity to this relationship. This suggests that the inflammatory response pathway plays a different role in carcinogenesis and cancer progression in HCC. Various factors, such as the tumor's ability to manipulate the immune response or changes in the tumor microenvironment, along with other immune-related and pro-cancerous signaling, are involved in inflammation and their counterbalance may affect patient outcomes. Such complexities in cancer has been reported in other pathways. For example, certain cellular pathways, like DNA repair, exert opposing effects during these two phases. While DNA repair mechanisms inhibit cancer initiation, they can also enable established cancer cells to resist cytotoxic therapies and perpetuate genetic instability. It is crucial to understand that inflammation plays an important role in both carcinogenesis and cancer progression. These two conditions are distinct from each other, and our method may prove to be very useful in comprehending this complex inflammatory status in cancer.

Our study is not without limitations. Given that this is a retrospective study utilizing various public patient data cohorts, some important clinical data, such as treatment details, were not accessible. The relevance of inflammatory response to drug treatment in HCC needs to be investigated in future studies. Furthermore, further studies are needed to understand the causal relationships and detailed mechanisms, as this study represents a single, "snapshot" observation.

## 5. Conclusions

Inflammatory response was associated with the HCC carcinogenesis sequence, and HCCs with a high inflammatory response correlated with immune cell infiltrations in the tumor microenvironment. These HCCs enriched immune responses as well as EMT, KRAS signaling, hypoxia and angiogenesis, and were associated with better patient outcomes.

## Data Availability

All the cohorts/datasets used in this study; The Cancer Genome Atlas (TCGA) and GO cohorts are all publicly available without any restrictions via cBioportal or Gene Expression Omnibus (GEO).

## Abbreviations

HCC: hepatocellular carcinoma
EMT: epithelial-to-mesenchymal transition
GSVA: gene set variation analysis
GSEA: gene set enrichment analysis
FDR: false discovery rate
NES: normalized enrichment score
DFS: disease-free survival
DSS: disease-specific survival
OS: overall survival
